# The Health Impact of Cocoa from Cultivation to the Formation of Biogenic Amines: An Updated Review

**DOI:** 10.3390/foods14020255

**Published:** 2025-01-15

**Authors:** Antonello Paparella, Maria Schirone, Clemencia Chaves López

**Affiliations:** Department of Bioscience and Technology for Food, Agriculture and Environment, University of Teramo, 64100 Teramo, Italy; apaparella@unite.it (A.P.); cchaveslopez@unite.it (C.C.L.)

**Keywords:** *Theobroma cacao*, cocoa-based products, cocoa production, processing, biogenic amines, health effect, food safety and quality

## Abstract

Cocoa and chocolate are known for their health benefits, which depend on factors like cocoa variety, post-harvest practices, and manufacturing processes, including fermentation, drying, roasting, grinding, and refining. These processing methods can influence the concentration and bioavailability of bioactive compounds, such as polyphenols that are linked to cardiovascular health and antioxidant effects. Recent scientific research has led to the development of cocoa-based products marketed as functional foods. However, despite the growing interest in the functional potential of cocoa, the literature lacks crucial information about the properties of different varieties of cocoa and their possible implications for human health. Moreover, climate change is affecting global cocoa production, potentially altering product composition and health-related characteristics. In addition to polyphenols, other compounds of interest are biogenic amines, due to their role and potential toxic effects on human health. Based on toxicological data and recent research on the complex relationship between biogenic amines and cocoa fermentation, setting limits or standards for biogenic amines in cocoa and chocolate could help ensure product safety. Finally, new trends in research on biogenic amines in chocolate suggest that these compounds might also be used as quality markers, and that product formulation and process conditions could change content and diversity of the different amines.

## 1. Introduction

Cocoa or cacao beans, obtained from the evergreen tree *Theobroma cacao* (family *Malvaceae*), are fermented and dried seeds, and represent the basic raw material used for manufacturing chocolate and cocoa derivative products, such as cocoa mass (paste or liquor), cocoa butter, and powder [1]. Although there are numerous genetic varieties, three commonly recognized species of *T*. *cacao*, i.e., Criollo (fine, delicate, and flavorful but susceptible to fungal diseases), Forastero (bitter and tart; common or ordinary taste, very resistant to climatic conditions and pests), and their hybrid Trinitario, with distinguished chemical composition, texture, and aroma, are the most appreciated and famous in terms of quality [2,3]. A fourth variety, known as Nacional, grows mainly in Ecuador, Mexico, and Venezuela, and has a complex aromatic profile, characterized by a floral aroma [4,5]. Differences in the sensory properties and physicochemical profile of the final product are due to cocoa variety, grain maturity, agrotechnical management, manufacturing process, and environmental conditions, such as geographical area, soil composition, and climate, in particular timing of sunlight and precipitation [6,7].

The cocoa tree is native to the Amazon region of South and Central America [8], and according to the Mayan, Inca, and Aztec civilizations it was considered “a gift from the gods” and used for both curative and nutritional purposes [9]. However, since 1961, the world leader in cocoa beans production has been Africa, especially West Africa [10], with a share in the year 2020/2021 equal to 77% of global production, followed by America (18%), and Asia/Oceania (5%). The top five cocoa producing countries include four West African states: Ivory Coast of Africa (2248 million tons in 2020/2021), Ghana, Cameroon, and Nigeria, where the tropical climate (average annual maximum temperature of 30–32 °C and an average minimum of 18–20 °C) is the most suitable for growing the cocoa tree [11], and one American state: Ecuador.

Table 1 shows global annual cocoa beans production from 2020 to 2024. According to the data, Ghana, the world’s second-largest producer, is the only country that has experienced a significant reduction in production. In the 2020/2021 season, production stood at 1047 million tons but by the 2023/2024 season, it had fallen to just 580 million tons. The International Cocoa Organization (ICCO) has predicted that cocoa production in Ghana and Ivory Coast will be 11% and 20% lower, respectively, compared to the 2022/2023 season. This decline in production, observed in both Ghana and Ivory Coast, is likely due to a combination of factors, including aging cocoa trees, severe drought linked to climate change, excessive rainfall, black pod disease [12,13], and internal challenges, such as the poor working conditions and the minimum cocoa price, which hinder economic progress for local inhabitants [14]. Cocoa prices have surged dramatically in recent years, reaching an all-time high by March 2024, doubling since 2022. This sharp increase, driven by supply chain disruptions and climate change, has created challenges for both consumers and the chocolate industry [15].

**Figure 1:**
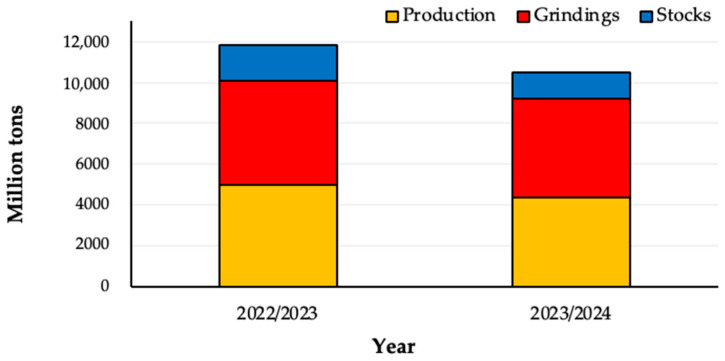
Cocoa production, grinding activities, and year-end stock [12].

The latest ICCO report highlights revised estimates for global cocoa production, grindings, and stocks for the 2022/2023 and 2023/2024 seasons. Both seasons faced supply challenges, resulting in a deficit. A decrease in global production, coupled with robust consumption, has led to a significant reduction in global cocoa ending stocks. For the 2023/2024 season, global cocoa production dropped significantly due to adverse weather, pests, and diseases in major West African producers, now estimated at 4382 million tons. Global grindings have reached 4816 million tons, though high cocoa prices and shortages have slowed processing activities, with demand continuing to exceed supply (Figure 1). According to ICCO projections, ending stocks for the 2023/2024 season are expected to be nearly 33% lower than those in 2020/2021. This decline has been a key factor driving the sharp increase in prices and volatility [16].

**Figure 2:**
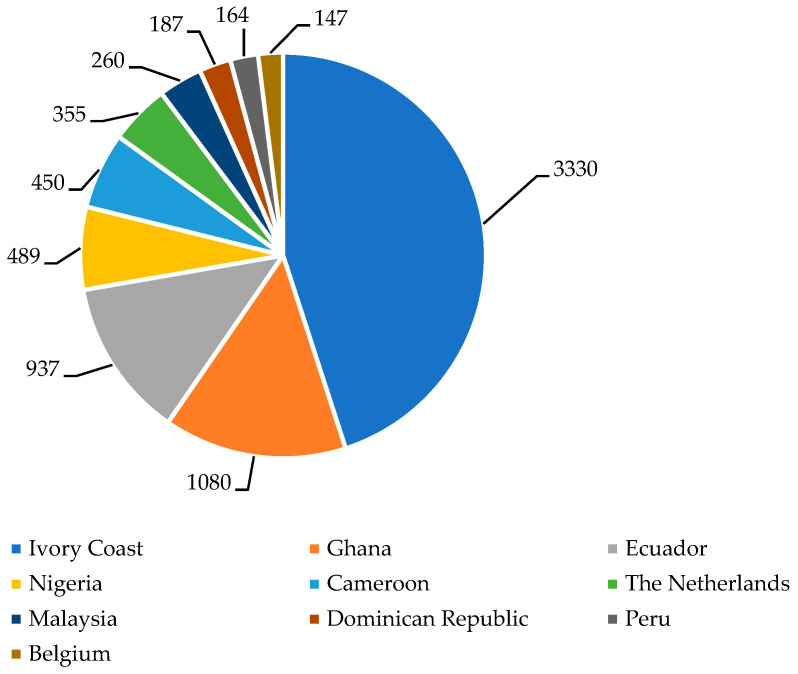
Top 10 cocoa-exporting countries globally. Data are expressed as export value (USD million) and are obtained from Observatory of Economic Complexity. The Netherlands and Belgium do not produce cocoa beans; they re-export the cocoa beans they import [18].

In 2022, The Netherlands (import value equal to USD 1.54 billion), the United States and Germany were the world’s first, second and third largest cocoa bean importing countries, respectively [17]. In 2022, cocoa beans were the 423^rd^ most traded product globally, with a trade value of USD 8.29 billion, marking a 20.3% decrease from the previous year’s value of USD 10.4 billion. The largest exporters were Ivory Coast (USD 3.33 billion), followed by Ghana (USD 1.08 billion), Ecuador (USD 937 million), Nigeria (USD 489 million), and Cameroon (USD 450 million) (Figure 2) [18].

Cocoa and cocoa-based products contain essential nutrients such as proteins, lipids, carbohydrates, and minerals, which positively impact health due to their antioxidant and anti-inflammatory properties [19]. However, excessive consumption may have detrimental effects on human health. Unfermented cocoa contains various free amino acids and biogenic amines (BAs), which change during fermentation and roasting processes. Some BAs exhibit benefits to well-being. For examples, polyamines—including spermidine and spermine—show antioxidant activity and help prevent cardiovascular diseases. Neuroactive compounds such as 2-phenylethylamine, tryptamine, and serotonin are also present. However, high concentrations of histamine and tyramine can have adverse effects, especially in sensitive individuals [20,21]. Gloria et al. [22] reported that unfermented Forastero cocoa from Bahia (Brazil) contained a total BA level of 30.87 mg/kg. During fermentation, the overall amine content—including spermidine, tryptamine, tyramine, and serotonin—decreased, while 2-phenylethylamine accumulated.

Oracz and Nebesny [23] found that tyramine was the most prevalent in raw cocoa beans, but roasted cocoa beans had higher levels of 2-phenylethylamine. Roasting increases BA concentrations, with temperature and humidity being key factors. These elevated levels may result from high-temperature treatments that convert precursor compounds into BAs. However, the exact mechanisms behind these changes during roasting still require further investigation. Furthermore, significant variation in BA concentrations has been observed across different cocoa beans varieties. This information could be useful for assessing BA intake from cocoa products and managing potential health risks.

The aim of this review was to describe and discuss the scientific literature on the impact of cocoa and cocoa products on human health, considering the characteristics of the process, the impact of fermentation, and the formation of BAs. While previous reviews have mainly focused on the functional properties of cocoa, this review gathered updated information on the effects of processing on health potential, as well as the significance of BAs as quality markers for cocoa products.

## 2. Cocoa Bean Processing

The conventional method used by most cocoa industries to transform the cocoa fruit into cocoa mass and derivatives is complex and involves in two main steps: preprocessing and processing. The preprocessing stage is carried out by the cocoa farmers and includes growing, harvesting, extraction, fermentation, drying, packing, and transporting cocoa beans. The cocoa tree’s fruit, known as pods, contains 20–50 beans or seeds depending on the type of cocoa tree [24]. To produce half a kilogram of chocolate, about 400 beans are needed [8].

Like other fermented foods, cocoa undergoes natural fermentation, and the final product is influenced by various local conditions, such as regional climates, cultural traditions, local preferences, availability of raw materials, and specific production methods unique to each growing area [25]. Immediately after manual harvesting, the fruits are cut open, and the cocoa beans are removed from the placenta and residual cocoa shell/pod elements. Then, the fresh beans and the surrounding pulp are subjected to spontaneous fermentation (from three to seven days based on the cocoa variety and environmental conditions), mainly in heaps or boxes piled up on the ground and covered with banana leaves [26,27].

During traditional fermentation, a combination of microbial and enzymatic reactions is carried out by the environmental “cocobiota”, described as a consortium of bacteria and fungi species [28]. Yeasts, lactic acid bacteria (LAB), and acetic acid bacteria (AAB) work in succession, breaking down the mucilage surrounding the seeds. In addition, cocoa proteins can be hydrolyzed during fermentation, either by two naturally occurring enzymes in the beans or by activity of naturally occurring microorganisms, resulting in the release of free amino acids, which can undergo microbial decarboxylation, leading to the formation of BAs [29]. During spontaneous fermentation, different compounds are produced that give the flavor to the final product and inhibit the growth of ochratoxigenic fungi (i.e., *Aspergillus niger*) [30,31].

Fermentation can be divided into three steps. The first phase (24–36 h) is controlled by anaerobic yeasts, which are tolerant to ethanol, heat, and acid, and convert fermentable sugars into ethanol, CO_2_, and H_2_O. The environment is characterized by pH < 4, low amount of oxygen and a temperature that can reach 35–40 °C due to the exothermic reaction in the production of alcohol. Carbohydrates and citrates are consumed, and after hydrolyzation of the pulp, the air enters into the cocoa pulp-bean mass, creating an aerobic environment that is favorable to LAB growth. The second step begins with the decline in yeast population. Although LAB exist from the beginning of fermentation, they dominate between 48 and 96 h after the start and produce lactic acid. During the third step (around 48–112 h), aeration rises, and AAB become active, generating an acidic environment for proteolysis and contributing to the development of the aroma precursors. The conversion of ethanol into acetic acid produces a temperature increase (around 50 °C or higher) [29,32]. In addition to AAB, other bacteria, i.e., *Bacillus* and *Enterobacteriaceae*, are found in the third step of the fermentation. The role of these microorganisms was investigated by Illeghems et al. [33], who suggested that *Enterobacteriaceae* can take up and metabolize the carbohydrates present in the cocoa pulp-bean mass, thus presumably having an important role in the overall metabolic processes of cocoa bean fermentation, e.g., for citrate assimilation and pectinolysis. On the other hand, *Bacillus* species play a significant role in cocoa fermentation, contributing to the development of flavor and aroma compounds while improving the microbiological quality of cocoa beans. Their antifungal properties help mitigate the production of undesirable compounds, enhancing the sensory attributes of chocolate [34].

The microbial association, considering not only the microorganisms deriving from the surfaces of the cocoa shell but also those originating from the banana or plantain leaves, insects or larger animals, agricultural equipment, residues from previous fermentation in boxes, etc., is activated by the variations of temperature during the process [35]. In these early steps of cocoa beans processing, contamination by microorganisms from the environment, personnel or utensils is very likely. However, some factors, such as changes in pH, water activity (a_w_), sugar content, and heat treatments, may favor resistant microbial species that best adapt to the specific environmental conditions [36].

Traditional fermentation is an empirical and uncontrolled process and therefore may be incomplete or give a final product with lacking or unpleasant flavors. To improve and control the quality and the progress of cocoa beans spontaneous fermentation, starter cultures can be added. As documented in the scientific literature, a combination of species, for example *Saccharomyces cerevisiae*, *Limosilactobacillus fermentum* (previously known as *Lactobacillus fermentum*), and *Acetobacter pasteurianus*, could represent a good opportunity to obtain a safe and controlled fermentation that enhance the aroma profiles of the cocoa beans [37]. Furthermore, *Kluyveromyces marxianus*, *Pichia kluyveri*, *Pichia kudriazevii*, *Hanseniaspora opuntiae*, *Torulaspora delbrueckii,* and pure cultures of *Lactiplantibacillus plantarum* (previously known as *Lactobacillus plantarum*) and *A*. *pasteurianus*, have proved to be successful starter cultures [38,39,40,41,42].

Biochemical changes during fermentation and roasting are crucial in the formation of peptides and free amino acids by endogenous peptidases that break down the proteins, and the reduction of sugars, which are precursors of the Maillard reaction during roasting. Polyphenols, one of the largest groups of compounds characterizing the plant kingdom (i.e., vegetables, tea, wine, etc.) undergo various reactions, such as oxidation and polymerization, leading to the formation of complex compounds. The polyphenol content can decrease significantly during the fermentation, roasting, and alkalizing processes, dropping from 100% to as low as 10%. This reduction results in a less bitter and astringent flavor in the final cocoa-based products [43,44]. Phenolic compounds are primarily stored in the cotyledons of the seeds, and if they diffuse out of cotyledons, polyphenol loss may occur [45]. Temperature and processing time are key factors driving this significant reduction [3]. During fermentation, the decrease is due to enzymatic activities and a rise in temperature (around 50 °C). The reduction in polyphenols is proportional to the degree of fermentation, with reported variations in antioxidant activity (e.g., 20–40% decrease) [46,47,48]. Additionally, sun drying causes a significant reduction in polyphenols up to about 70%, depending on the season and processing conditions. Drying at higher temperatures or using different methods also leads to polyphenol loss. Roasting, particularly at temperatures of 120–150 °C, significantly reduces polyphenols (with losses ranging from 0% to 95%, depending on the temperature, time, and cultivar). High temperatures accelerate polyphenol degradation, although some variations occur depending on the specific roasting conditions. Antioxidant activity is also depleted during roasting, with reductions ranging from 37% to 50% [49]. A decrease of the flavan-3-ol content, monomeric (-)-epicathechin, and total anthocyanins was also reported [50].

Fermentation and roasting, key steps in defining cocoa quality, flavor, and texture, involve temperatures ranging from 110 °C to 160 °C for 15–40 min [51,52]. After fermentation, the moisture content of the cocoa beans needs to be decreased from 55% to 7.5% to ensure temporary storage in the tropical environment, before shipping to the cocoa factories. Drying can be carried out by artificial dryers in a warehouse or by using solar energy in the open air, although the risk of contamination from the environment or wild animals can be a very important problem. Using the artificial system, the drying time decreases, and meanwhile relative humidity is low; a temperature of 30–40 °C, like the thermal conditions obtained with solar drying, remains high and constant [53]. It is also possible to use wood fires in a space below the drying area; in this case, heat passes through a chimney, and the smoke that comes out can give the beans an unpleasant and acidic taste [54].

**Figure 3:**
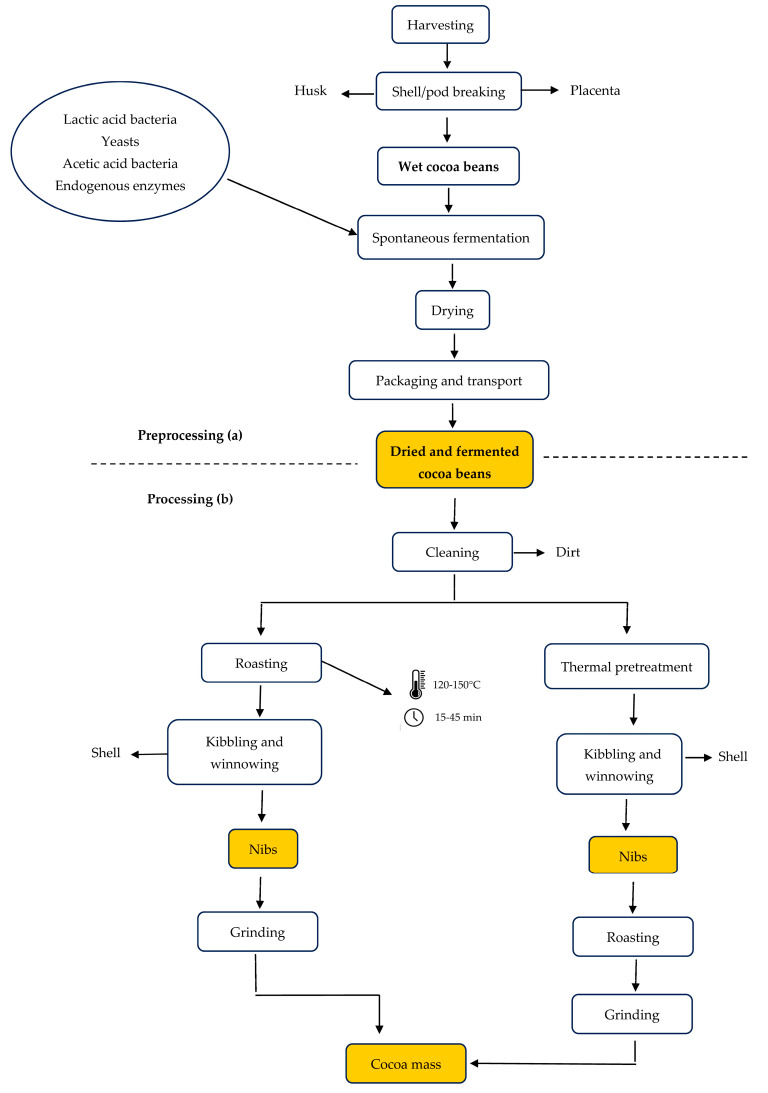
Preprocessing (**a**) and processing (**b**) phases of cocoa beans [45,58].

In the cocoa factories, processing starts with cleaning and inspection of the dry cocoa beans to remove foreign materials. At this point, some industries can apply a thermal pretreatment to obtain cocoa beans with a similar size, followed by kibbling and winnowing, and only then roasting. This last step is one of the main technological operations in the processing of cocoa beans, where temperatures ranging from 120 °C to 150 °C for 15–45 min are used to reduce and deactivate microorganisms, obtaining a final product with a longer shelf life and specific characteristics in color, taste, and smell [55,56]. The shells, which are exposed to undesirable contaminants, are removed from the rest of the beans through winnowers. Then, immediately after roasting and shelling, the nibs (beans without the outer shell) are ground to obtain the cocoa mass, also known as cocoa liquor or cocoa paste [8]. In Figure 3, cocoa bean preprocessing and processing steps are schematized.

When the cocoa liquor is mechanically pressed, it separates into cocoa butter and cocoa powder, which is obtained by grinding/sieving the cocoa cake. Cocoa liquor is solid at room temperature and is used to produce dark and milk chocolate with the addition of cocoa butter and sugar. According to the Directive 2000/36/EC [57] on cocoa and chocolate-based products, the following requirements are set in the European Union:

- cocoa butter contains *not more than 1.75% free fatty acid content (expressed as oleic acid) and not more than 0.5 except in the case of press cocoa butter, where it shall not be more than 0.35% unsaponifiable matter (determined using petroleum ether);*
- cocoa powder contains *not less than 20% cocoa butter, calculated according to the weight of the dry matter and not more than 9% water;*
- chocolate contains *not less than 35% total dry cocoa solids, including not less than 18% cocoa butter and not less than 14% of dry non-fat cocoa solids.*

Three types of chocolate are mainly produced and consumed in the world, characterized by a high market value: (i) black or dark, also known as plain chocolate, made from cocoa butter, sugar, cocoa liquor, emulsifier (i.e., lecithin), and vanilla essence; (ii) white chocolate, produced with cocoa butter, sugar, milk, and flavorings; (iii) milk chocolate, which is the same as dark chocolate with the addition of milk or milk powder.

## 3. Chemical Composition of Cocoa Beans

**Figure 4:**
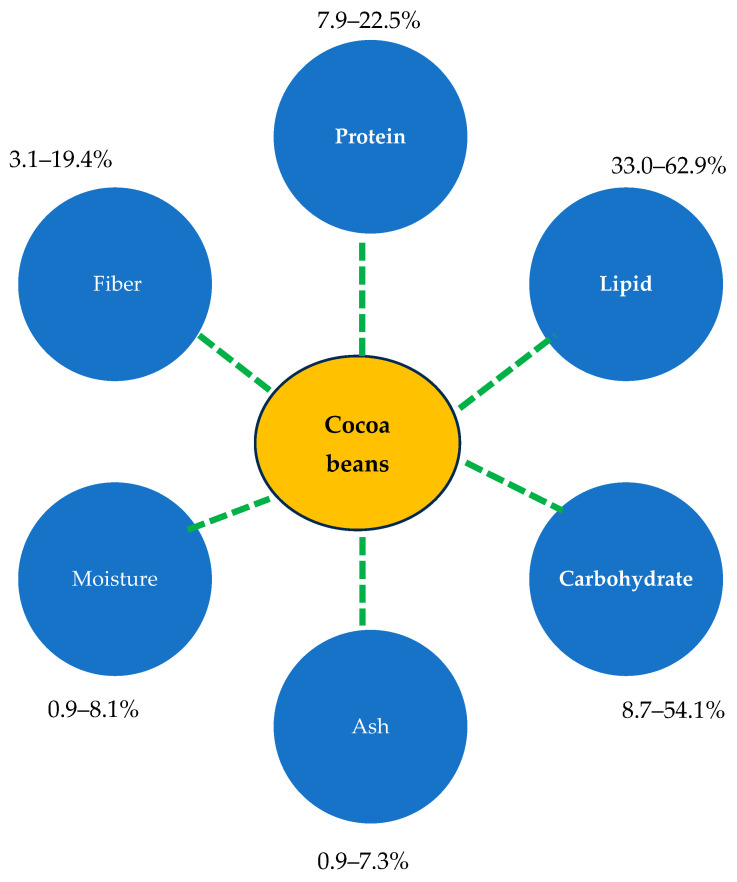
Chemical composition of unfermented and fermented dry cocoa beans [63].

The composition of cocoa beans is highly variable and is influenced by factors such as genetic type, geographical origin, and post-harvest processes including fermentation, drying, and packing [59,60,61,62]. On average, lipids are the main nutrient with a value ranging from 33% to 63%, followed by carbohydrates (about 9–54%) and proteins (about 8–23%) (dry basis). The moisture and ash content are less than 10% (Figure 4).

Triacylglycerols represent 97–98% of the lipids in cocoa beans, contributing to the texture, taste, and appearance of cocoa products. These fats are primarily composed of palmitic (24.1–27.1%), stearic, and oleic acids (approximately 32.7–37.6% for both), with small amounts of linoleic acid. The specific fatty acid profile influences the consistency and sensory properties of cocoa products, such as their hardness and flavor [64].

Cocoa beans contain proteins predominantly in the form of albumin (52%) and vicilin (7S)-class globulin (43%), with smaller amounts of prolamin and glutenin. During fermentation, two endogenous enzymes, aspartic endoproteases and carboxypeptidases, are activated, leading to protein breakdown and the formation of free amino acids and oligopeptides. These compounds, including hydrophobic amino acids, such as leucine, alanine, valine, phenylalanine, contribute to the development of cocoa’s characteristic aroma, including aldehydes and pyrazines [65,66]. BAs can accumulate and increase during fermentation, primarily through the microbial decarboxylation of amino acids as well as the oxidative decarboxylation of amino acid precursors due to thermal processes [67]. At low levels, BAs are relevant to human health for their biological activities (i.e., cardiovascular protection, mood modulation, antioxidant activities, etc.), but if present in significant quantities in foodstuffs and/or ingested in the presence of potentiating factors, they can lead to undesirable physiological and toxicological effects [68,69].

Sucrose (around 90% of total sugars), fructose and glucose (both 6% of total sugars) are the polysaccharides mostly contained in cocoa beans, followed by low quantities (less than 0.5 mg/g) of sugar alcohols, such as mannitol and inositol [70,71]. The enzyme invertase breaks down sucrose into reducing sugars, which play a role in the Maillard and Strecker reactions during roasting [7], leading to the formation of flavor compounds like pyrazines, aldehydes, and esters responsible for fruity, floral, and nutty notes [72,73].

Many minerals, particularly potassium, magnesium, phosphorus, and calcium, are the most abundant in cocoa beans, whereas sodium, copper, zinc, and iron are present in small amounts [74,75]. Mineral components are essential for the vascular function and the optimal functioning of all biological systems. For example, magnesium and calcium are macro-elements that play a significant role in the human body, including the regulation of blood pressure, muscle function, blood glucose control, and nerve transmission [76,77].

Cocoa beans also contain significant levels of vitamins, particularly vitamin D, B1, B2, and E, mostly as γ-tocopherol [58,78]. Kühn et al. [79] observed that cocoa beans can be contaminated by fungi containing substantial concentrations of ergosterol, a precursor of vitamin D_2_, and suggested that ergosterol might be converted to vitamin D_2_ during drying. The same authors found that high amounts of vitamin D_2_ were present in cocoa powder and butter manufactured by cocoa beans originating from diverse growing regions. Concerning chocolate, dark and white chocolate had the highest (1.90–5.48 μg/100 g) and the lowest (0.19–1.91 μg/100 g) quantities of vitamin D_2_, respectively.

Moreover, cocoa is a good source of dietary fiber including cellulose, hemicellulose, and pectin substances [80]. Due to the presence of fibers, cocoa products are considered an attractive and palatable food from a probiotic point of view [81].

*Theobromine and Caffeine*

Cocoa contains methylxanthines, predominantly theobromine or 3,7-dimethylxanthine, and in smaller quantities, caffeine or 1,3,7-trimethylxanthine (about 4% and 0.6% on a fat free basis, respectively), as well as theophylline (1,3-dimethylxanthine) in quantities that are often considered negligible [82,83,84,85]. These compounds are psychoactive and contribute to cocoa’s characteristic bitter taste [86,87,88,89].

Caffeine, like theobromine, is an antagonist of adenosine receptors and is known as a psychostimulant. While cocoa contains less caffeine than coffee, tea, and soft drinks, it still has notable neuroprotective effects, potentially contributing to the prevention of neurodegenerative diseases like Alzheimer’s and Parkinson’s [90], as well as providing benefits such as improved concentration and reduced fatigue [91].

*Polyphenols*

Cocoa beans are an excellent source of phenolics or polyphenols, particularly flavanols (catechin and epicatechin), anthocyanidins, and proanthocyanidins, which account for 37%, 4%, and 58% of the total polyphenol content, respectively [92,93,94]. Fresh cocoa beans are inedible due to their high polyphenol content, which results in a bitter aroma and an unpalatable and unpleasant flavor. However, during processing, the concentration and proportion of these bioactive compounds change, leading to a reduction in acidity and astringency [95,96,97].

Polyphenols are valuable not only for their antioxidant effects and sensory contributions [98], but also for their nutritional and health benefits [99]. Heiss et al. [100] reported that the consumption of cocoa drinks containing high levels of flavan-3-ols can lead to a reduction in endothelial dysfunction through an increase in nitric oxide bioactivity. Baba et al. [101] evaluated the levels of epicatechin and its metabolites in 25 healthy, normal weight and non-smoking subjects. These authors found that the consumption of 26 g/d for 12 weeks of cocoa powder may lead to a significant increase in plasma HDL-cholesterol concentrations by providing suppression of LDL oxidation. The same results were observed in another study performed on 160 normocholesterolemic and mildly hypercholesterolemic individuals who consumed different amounts of cocoa powder (13, 19.5, and 26 g/d for low-, middle-, and high-cocoa groups, respectively) for four weeks [102].

Rusconi and Conti [94] stated that the human body can quickly adsorb epicatechin, which is measurable in the plasma as early as 30 min after intake, reaching its peak 2–3 h after chocolate consumption and returning to the baseline value within 6–8 h [103]. Therefore, it is possible that flavanols and their metabolites may enter particular areas of the brain linked to memory and learning, which suggests a strong positive impact on cognition and neuroprotection [104]. Grassi et al. [105] observed that short-term consumption (five treatments lasting one week each) with a daily intake of 10 g cocoa (0–800 mg flavonoids/day) by 20 healthy non-smoker subjects aged 18–70 years led to an improvement of pro-inflammatory mediators, oxidative stress, and lipid peroxidation. The effect was significant when doses of flavonoids were higher. The intake of flavonoids has also been associated with a reduced risk of cardiovascular disease and some types of cancer [106]. Such functional properties have been demonstrated both in healthy people and in individuals with risk factors (i.e., hypertension, smoking, overweight and obesity, diabetes, or hyperlipidemia). In particular, the consumption of foods that are rich in polyphenols can help reduce the onset of atherosclerotic lesions [107]. According to the scientific evidence published by the EFSA Panel on Dietetic Products, Nutrition and Allergies, cocoa flavanols “help maintain the elasticity of blood vessels, contributing to normal blood flow”; this effect is obtained if “200 mg of cocoa flavanols is consumed daily, provided by less than one gram of high-flavanols cocoa extract in capsules or tables, in the context of a balanced diet”. This amount can be obtained by eating 2.5 teaspoons with high-flavanol cocoa powder or 10 g of dark chocolate [108].

## 4. Health Benefits and Potential Risks of Cocoa Products

Cocoa and its derivates are not only prized for their flavor and sensory qualities but also for their health-promoting properties. Many scientific reports are available about the multiple benefits of cocoa, such as high antioxidative activity [109], positive impact on skin health [110], free radical scavenging [111], lowering blood pressure [112,113], vascular function and cardiovascular outcomes [114], improvement of glucose metabolism [115,116], and inhibition of platelet aggregation and adhesion [117].

Due to its significant impact on human health, cocoa can be considered a functional ingredient capable of enhancing bioactive characteristics in foods to which it is added. The term “functional food” refers to foods containing active compounds in sufficient quantities that provide beneficial physiological effects to human health beyond basic nutrition [118]. Consumption of cocoa-based products has grown significantly over the years, and while the global cocoa market was approximately USD 48 billion in 2022, it is expected to reach USD 79 billion by the end of 2032, increasing at a CAGR (compound annual growth rate) of 5% (2022–2032) [119]. Research on functional chocolate has been increasing in recent years, and several products are at the level of patents, with China as a leading country [120]. Recent publications have investigated the fate of bioactive compounds during chocolate processing and in vitro gastrointestinal digestion [121], as well as the effect of milk powder in enhancing the digestibility of functional chocolate [122]. Very recently, chocolate products named “functional” or “bioactive” have been launched on the market [123], although this claim would need to be documented with in vitro, in vivo, and clinical studies. In this respect, Balcázar-Zumaeta et al. [118] have proposed to classify functional chocolates into three categories: functional chocolates, potentially functional chocolates, and chocolates enriched with bioactive compounds.

All in all, cocoa is a very versatile ingredient, used in food, and cosmetic, pharmaceutical, and agricultural products, where it adds important functional properties [124]. In the near future, cocoa and in particular cocoa shell might also become a strategic source for the production of energy or biofuels [82]. Moreover, cocoa shell exhibited prebiotic activities when added to ice cream, while cocoa in oral products demonstrated anticariogenic and antibacterial capacities [125,126]. Functional properties have also been demonstrated in different cocoa by-products, such as pod husk and pulp [127]. There is a growing body of evidence that the functional properties of cocoa products could be strategic in maintaining health. In fact, cocoa in the diet might play an important role in influencing gut homeostasis [128], by interacting with the gut microbiota [129]. However, on balance, further studies are needed to fully characterize the potential actions of cocoa compounds on dietary metabolic disorders and the gut microbiota [84].

For many years, cocoa and its derivatives, and in particular chocolate, have been consumed as confections, aphrodisiacs, and medicines for the treatment of various syndromes, such as depression, mental disorders (memory, attention, verbal fluency), flu conditions, fertility problems, and digestive difficulties. In fact, in several studies, much attention is paid to chocolate as a “food for moods” [130,131,132]. Chocolate is usually consumed by all age individuals for its pleasant sensory properties as an antidepressant linked to emotional comfort. For its antidepressant effects, chocolate has been described as “the Prozac of plants” [133]. The wellness effect is attributed to neuroactive BAs, such as 2-phenylethylamine, tryptamine, and serotonin, which play a role in mood modulation, as well as dopamine, a neurotransmitter involved in the regulation of human behavior. However, on the negative side, histamine and tyramine can have adverse effects in sensitive individuals.

## 5. Occurrence and Role of Biogenic Amines in Cocoa Products

**Figure 5:**
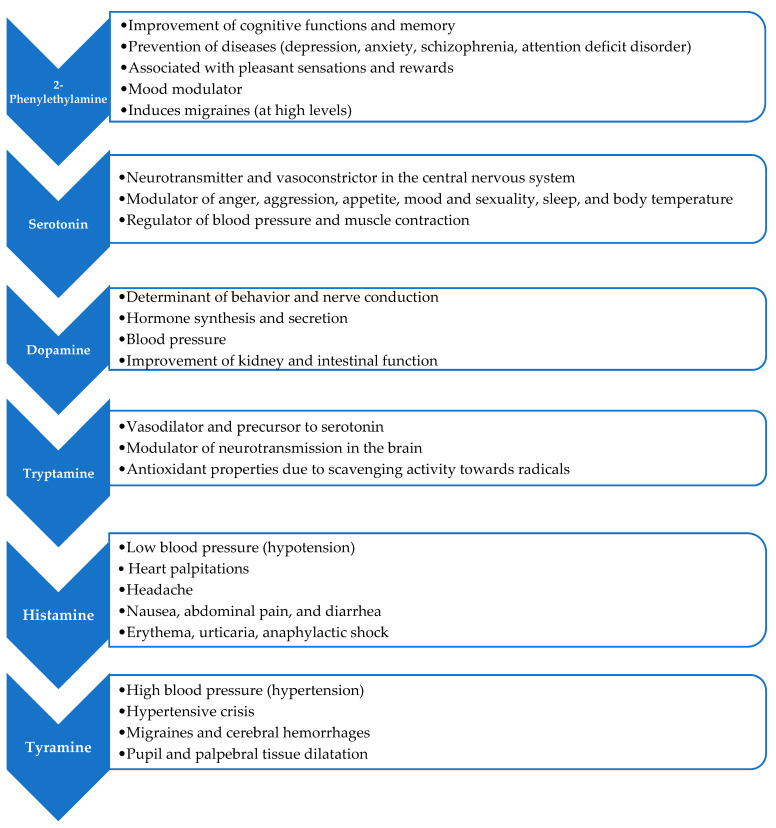
Main effects of the most common BAs linked to cocoa and its derivates on human health.

BAs serve important roles in plant development as hormone precursors and in defense against predators [134]. In humans, BAs can have both positive and negative health effects [135,136]. In Figure 5, the main properties of some common BAs are reported. Among the various BAs found in cocoa and chocolate, tyramine and histamine are of particular concern due to their potential toxic effects. Tyramine can cause hypertension, headache, increased blood pressure, and pupil and palpebral tissue dilatation [137], with a no adverse effect level (NOAEL) established as 600 mg tyramine per person per meal for healthy individuals not taking monoaminoxidase inhibitor (MAOI) drugs. However, the daily dose of tyramine should be decreased to 50 mg for individuals taking third-generation MAOI drugs and 6 mg for those taking classical MAOI drugs [138]. Instead, high levels of histamine can cause hypotension and other cardiovascular symptoms, headache, nausea, and abdominal pain, with a NOAEL of 50 mg histamine per person per meal for healthy population, but below detectable limits for individuals with histamine intolerance [139]. Tyramine can also be released from chocolate during in vitro intestinal digestion, raising concerns about hypertensive crises or migraines, especially in sensitive individuals [68]. Both tyramine and histamine can be particularly hazardous for individuals with gastrointestinal conditions, genetic predispositions, or those using certain medications [140,141].

Other BAs, such as putrescine and cadaverine, are generally undesirable in chocolate because they can contribute to off flavors [139] and interfere with metabolism of other amines, thereby exacerbating their toxicity [29]. Additionally, these amines can interact with nitrites to form nitrosamines, which are carcinogenic [142].

On a more positive note, spermidine and spermine—amines present in all living cells—have antioxidant properties that protect against cell membrane and DNA damage. At physiological levels, they are efficient scavengers of hydroxyl radicals and are associated with the reduction of blood pressure and occurrence of cardiovascular diseases [143].

Climate change poses a significant threat to food systems, with implications for both food safety and the quality of cocoa products. Rising temperatures and altered rainfall patterns can lead to regional and varietal differences in the concentration of BAs. These climate changes can also disrupt the fermentation process, which is crucial for developing the distinctive flavor of cocoa. Temperature fluctuations affect the microbiota involved in fermentation, including yeasts, bacteria, and filamentous fungi, which can impact the formation of BAs [144]. Increased temperatures may accelerate the breakdown of amino acids into BAs [145], altering the flavor and aroma of cocoa products and potentially compromising their safety. Thus, understanding the relationship between climate change, cocoa cultivation, and BAs formation is essential to maintaining the quality and safety of chocolate products in the face of global warming. As the global climate changes, there will likely be increasing challenges in managing BA concentrations to ensure the quality and safety of cocoa-based products. Table 2 presents data on some BAs in different cocoa products.

## 6. Formation of Biogenic Amines During Cocoa Fermentation

Fermentation of cocoa is significantly influenced by the microbial strains involved, which in turn affect the levels of BAs. LAB strains involved in food fermentations are recognized as microorganisms of a low food safety risk, even though their metabolism activity could lead to the accumulation of BAs. For example, *Lactiplantibacillus fabifermentans* and *Furfurilactobacillus rossiae* enhance proteolytic processes, leading to a richer free amino acid profile in cocoa, which can subsequently lead to higher BA production [152]. Recent studies have also highlighted the potential for yeasts and filamentous fungi in cocoa fermentation to contribute to BA formation. Delgado-Ospina et al. [29] reported that 65 out of 165 yeast strains isolated from fermented cocoa beans were found capable of decarboxylating in vitro at least one of the tested amino acids involved in the formation of the most important BAs in cocoa, such as cadaverine, histamine, spermidine, and spermine. In particular, the strains of *S. cerevisiae, P. kudriavzevii*, *Pichia manshurica*, *Zygosaccharomyces bisporus*, *Candida parapsilosis*, *Schizosaccharomyces pombe*, *Trichosporon asahii* var. asahii, and *Wickerhamomyces anomalus* were the most active. In the same way, filamentous fungi, and namely *Aspergillus niger*, *Aspergillus flavus*, *Aspergillus tamarii*, *Penicillium citrinum*, and *Byssochlamys spectabilis*, were found to be potential BAs producers [153]. However, the generation of BAs by decarboxylase-positive strains, evaluated in vitro, does not inherently suggest analogous behavior in cocoa fermented beans, as the accumulation of BAs is influenced by a plethora of factors (including temperature, pH, availability of precursor amino acids, fermentation duration and temperature, among others) and their interactions [154]. Indeed, the decrease in pH and the concomitant increase in total titratable acidity that occur during fermentation catalyze the production and liberation of free amino acid decarboxylases from certain microorganisms, thereby facilitating the formation of amines [144,155]. On the other hand, during fermentation, a fluctuating pattern of BA content was observed in conjunction with the rise in temperature and duration of fermentation, likely attributable to the interplay between the enzymatic activities of amino acid decarboxylases and amino oxidases, which respectively lead to the accumulation and degradation of BAs [29].

The accumulation of BAs throughout fermentation exhibits variability depending on the specific methodology employed, microbial strains involved, and ambient temperature; consequently, this phenomenon justifies the attribution of distinct functional properties and sensory characteristics to chocolate. In this context, do Carmo Brito et al. [46] reported for the first time alterations in the BAs profile of the Forastero hybrid cultivar. They proposed a classification of the fermentation process of cocoa beans into three distinct stages: (i) during the anaerobic fermentation phase, both unfermented and fermented beans up to three days exhibit high concentrations of tryptamine, phenolics, and scavenging capacity; (ii) from day four until the midpoint of fermentation, elevated levels of spermidine, total BAs (sum of tryptamine + tyramine), polyamines (sum of spermidine + spermine), and total bioactive amines (sum of BAs + polyamines) are observed; and (iii) at the conclusion of fermentation, the highest concentrations of SPD and total acidity are recorded. Nevertheless, they did not establish any correlation with the specific microbial groups involved. In contrast, Deus et al. [140] found that during Forastero cocoa fermentation, spermidine, tryptamine, tyramine, and putrescine were consistently present, while serotonin was detected only up to 72 h, and phenylethylamine appeared after 60 h. The concentrations of individual amines and the total amine content varied considerably throughout the fermentation process, peaking between 96 and 120 h. Spermidine was the most abundant amine in the final product, followed by phenylethylamine, tyramine, putrescine, and tryptamine.

The fermentation technique used can also impact BA levels. Ganeswari et al. [156] indicated that the fermentation of cocoa beans in shallow containers lined with banana leaves leads to a reduction in bean acidity and potentially lower concentrations of BAs. Furthermore, recent findings by Silveira et al. [134] elucidated that the specific variety of cocoa significantly affects the levels of BAs present in cocoa and chocolate products. For example, PS 1319 variety exhibited elevated levels of cadaverine in comparison to Parazinho variety. Conversely, it has been noted that various microbial strains display differing abilities to either produce or degrade BAs, which subsequently impact the safety and quality of the final product. For instance, *S. cerevisiae* and *L. fermentum* were found to elevate overall BAs levels during fermentation [157], while certain yeast strains, such as *P. kudriavzevii* and *S. cerevisiae,* exhibited notable reductions in cadaverine and 2-phenylethylamine, while concurrently increasing levels of tryptamine and putrescine [149]. Certain LAB strains can inhibit BAs accumulation, suggesting that a careful selection of starter cultures can mitigate risks associated with BAs [137,158].

Recently, Silva et al. [68] investigated modulating the ratios of the different BAs in chocolate by adding different levels of underfermented beans during processing. In this study, the authors found that histamine and spermidine were not affected, whereas tyramine, agmatine, 2-phenylethylamine, and serotonin were higher in chocolate with ≤20% under-fermented cocoa. Moreover, if ≥35% under-fermented cocoa beans were added during chocolate processing, serotonin and agmatine could not be detected after in vitro digestion. Given the lack of information regarding the BAs profiles of different cocoa varieties and their potential impact on human health, this study opens new perspectives for precision processing aimed at reducing tyramine levels in chocolate products.

## 7. Limits and Standards for Biogenic Amines in Cocoa Products

When it comes to proposing any limit for BAs in foods, it should be considered that the total content of BAs in the human body is the sum of endogenous BAs, formed by human cells, and exogenous BAs, derived from food and gut microbiota. Among BAs, only histamine is considered at a regulatory level, with limits established only for fish and fish products in the European Union and in the United States of America. However, in terms of public health, it might be important to define limits for histamine in other food categories, as well as for tyramine and other BAs. In fact, beyond histamine and tyramine, toxicological data are available for other BAs. For example, putrescine, whose ingestion at high levels could cause hypotension, bradycardia, and limb paralysis [159], can be considered a health risk at a concentration of >880 mg kg^−1^ of food [138], while a limit of 180 mg kg^−1^ has been suggested for cheese, which is the food with the highest concentration of putrescine [139]. Less information is available on the toxicity of cadaverine, which has been associated with putrescine-like adverse effects; it has been suggested that food concentrations of >510 mg kg^−1^ could have toxic effects [142]. Although toxicity of BAs in laboratory animals and in cell lines is documented in scientific literature, it is clearly difficult and dangerous to extrapolate legal limits or even standards for foods from results obtained in laboratory conditions. Considering that several different food categories contribute to the overall BAs intake, a maximum total amount of BAs in food of 750–900 mg kg^−1^ has been proposed [160].

On the other side, BAs have also been proposed as a quality marker for cocoa-based products, based on the lower total amount found in organic and fair-trade products, compared to conventional counterparts [149]. However, to use BAs as a quality indicator, an extensive data collection should be performed, and the resulting values should be incorporated into voluntary standards or widely accepted guidelines. Some manufacturers such as Frusano in Germany have launched chocolate products with the claim “histamine-free”, based on regular and voluntary sampling, which is far from being a scientifically-sound standard for chocolate. In fact, to establish scientifically based limits or standards for BAs in cocoa products, it will be necessary to carry out risk assessment studies. These should include the evaluation of BAs content in various products under different processing conditions, as well as the quantity of cocoa products typically consumed in a normal diet.

## 8. Conclusions

Climate change is partially responsible for the decrease in global cocoa production and is likely to affect the characteristics of cocoa and its impact on human health. As a result, the future cocoa market will have to focus on quality rather than quantity, minimizing wastes and upgrading the functional value of finished products. At the same time, due to rising energy prices, cocoa and chocolate production needs to be rethought in terms of lean processing and precision technologies, such as controlling fermentation performance and managing roasting effects. In this context, manufacturers will have to navigate through difficult challenges, due to the lack of knowledge available on potential changes in the composition of the different cocoa varieties, and their subsequent impact on product quality and safety.

Technology forecasting data indicate that future changes in the chocolate market could be oriented towards functional products. These products are already present in the market, although their health impact needs to be assessed. From this viewpoint, future research needs to address important issues regarding both beneficial and adverse effects on human health. In this respect, BAs represent one of the research areas that requires more efforts and investments, considering the lack of risk assessment studies and the complex relationship between fermenting microorganisms and BAs content and composition. BAs could also be an interesting testing ground for precision technology, aimed at defining formulation and process conditions to increase functional properties, at the same time decreasing the content of undesired BAs. To reach these goals, it will be important to combine the experience of researchers in different fields, including food technology, microbiology, biochemistry, and toxicology.

## Figures and Tables

**Table 1:** Global annual production of cocoa beans, expressed in million tons, and average cocoa prices worldwide [12,15].

Countries	Year			
	2020/2021	2021/2022	2022/2023	2023/2024
Ivory Coast	2248	2121	2241	1800
Ghana	1047	683	654	580
Other Africa	761	785	774	788
Americas	935	973	1061	1035
Asia and Oceania	254	265	266	247
**World total**	5245	4827	4996	4450
Average Price *	2.43	2.39	3.20	4.40

Legend: * expressed in USD/Kg; data for 2022/2023 and 2023/2024 are estimates.

**Table 2:** Concentration range of some BAs in various cocoa products.

Reference	Samples	Biogenic Amines (mg/kg)						
		CAD	HIM	PEA	PUT	SPD	TYM	Total Content
Granvogl et al. 2006 [146]	Raw cocoa beans	0.2–1.2						
	Roasted cocoa beans	0.6–10.2						
	Dark chocolate							
Kosman et al. 2007 [147]	Grated cocoa	2.8–14.9					9.6–71.7	
Oracz and Nebesny 2014 [23]	Raw cocoa beans							2.7–11.4
	Fermented and dried cocoa beans			1.7–18.4			1.0–7.4	4.4–33.5
Baranowska and Płonka 2015 [148]	Chocolate 90%						47.6	
	Cocoa						46.8	
	Broad bean						22.6	
	Bean						31.0	
Restuccia et al. 2016 [149]	Cocoa-based products	nd–5.3	2.4–38.1	nd–2.0	nd–32.7	nd–9.7	1.3–31.7	5.7–79.0
	Cocoa powder	0–6.1	2.2–23.8	0–3.2	0–10.1	1.0–10.2	1.3–13.1	5.7–72.3
	Dark chocolate	0	2.9–35.0	0–1.1	0.9–6.1	1.0–9.8	1.6–12.4	7.7–65.0
	Milk chocolate	0	7.3–45.4	0	0.8–3.9	1.0–6.1	1.8–19.6	12.4–68.7
	White chocolate	0	9.5–38.1	0	0	2.0–6.2	3.0–20.6	16.4–75.3
	Powder to prepare cocoa drink	0	9.8	0	0.9	0	7.9	20.3
	Powder to prepare cocoa mousse	0	25.1	0	13.3	7.4	19.7	71.5
	Powder to prepare cocoa pudding	0	15.0–23.4	0	3.7–10.1	3.6–4.9	12.9–31.7	38.0–79
do Carmo Brito et al. 2017 [46]	Fermented cocoa beans	-	-	-	-	3.3–19.4	2.2–11.8	12.8–39.6
Spizzirri et al. 2019 [50]	Fermented and roasted cocoa beans	1.1–2.6	3.1–12.8	nd–2.1	1.5–10.9	nd–9.1	nd–11.1	12.9–58.3
Delgado-Ospina et al. 2020 [150]	Fermented and dried cocoa beans	nd–66.6	nd–41.9	nd	nd	0.3–48.7	nd–0.2	
	Roasted cocoa beans (120 °C for 22 min)	nd–5.5	nd–59.8	10.4–18.8	nd–6.5	nd–2.6	9.6–26.5	
	Roasted cocoa beans (135 °C for 15 min)	nd–5.5	nd–17.1	6.0–26.2	nd–62.6	nd–2.6	0.8–16.9	
Deus et al. 2021 [140]	Dark monoclonal chocolates	0–8.2				2.7–8.9	0.6–10.4	
Tuenter et al. 2021 [151]	White chocolate						<0.3	
	Ruby chocolate						<0.3	
	Milk chocolate						1.0	
	Dark chocolate						2.8	
Silva et al. 2023 [68]	Chocolate	2.3–7.2	0.6–1.2	0.8–2.2	2.3–5.7	3.2–5.6	1.4–2.8	14.9–34.7
Silveira et al. 2023 [134]	Roasted cocoa beans	nd–18.0		nd–2.5	nd–10.5	nd–5.7	nd–4.3	
	Liquor	nd–5.2	nd–0.6	nd–2.6	nd–4.4	nd–1.5	nd–3.9	
	Chocolate	nd–6.5	nd	nd–1.5	nd–4.0	nd–2.0	nd–3.6	

Legend: CAD = cadaverine; HIM = histamine; PEA = 2-phenyethylamine; PUT = putrescine; SPD = spermidine; TYM = tyramine; nd = not detected or below the limit of quantification.

## Data Availability

No new data were created or analyzed in this study. Data sharing is not applicable to this article.
